# Balancing Public Health and Economic Interests Whilst Creating New Opportunities for Labor Migrants

**DOI:** 10.1007/978-3-030-65355-2_28

**Published:** 2021-03-20

**Authors:** Conny Rijken

**Affiliations:** 1 Tilburg University, Tilburg, The Netherlands; 2 Tilburg University, Tilburg, The Netherlands; 3 Tilburg University, Tilburg, The Netherlands; 4 Tilburg University, Tilburg, The Netherlands; Department of Criminal Law, Tilburg Law School, Tilburg, The Netherlands

## Abstract

The COVID-19 pandemic unveils structural weaknesses and vulnerabilities in societal structures that we have become to take as ordinary parts of our society. This especially holds true for such structures in the labor market in general (see Chap. 10.1007/978-3-030-65355-2_5 by Bekker) and especially for labor migrants, the focus of this chapter. The COVID-19 crisis not only augmented their precarious situation but also enlarged the awareness of the dependency of developed countries on migrant workers and, in some countries, led to a positive response by way of regularization of the migratory status of migrant workers. Apparently, the COVID-19 crisis has revealed that a public health risk generated more impact than academic and grounded research on work and living conditions of migrant workers and the work of organizations fighting for migrant workers’ rights. In this chapter, I will first address how COVID-19 has impacted the position of migrant workers before discussing opportunities created for migrant workers and the way forward.

The COVID-19 pandemic unveils structural weaknesses and vulnerabilities in societal structures that we have become to take as ordinary parts of our society. This especially holds true for such structures in the labor market in general (see Chap. 10.1007/978-3-030-65355-2_5 by Bekker) and especially for labor migrants, the focus of this chapter. Over the past decades, rigorous scholarly work contemplated the precarious working conditions of migration workers due to labor market flexibilizations and avoidance of labor laws that have, in turn, received limited political attention and little willingness of industries and corporations to change these practices (Costello and Freedland 2014; Anderson and Ruhs 2010; Rijken and de Lange 2020). The COVID-19 crisis not only augmented these situations of precariousness but also enlarged the awareness of the dependency of developed countries on migrant workers and, in some countries, led to a positive response by way of regularization of the migratory status of migrant workers, e.g., in Italy. Interestingly, risks of COVID-19 outbreaks among migrant workers exposed working and living conditions that were known but neglected for too long. Apparently, the COVID-19 crisis has revealed that a public health risk generated more impact than academic and grounded research on work and living conditions of migrant workers and the work of organizations fighting for migrant workers’ rights. In the remainder of this chapter, I will first address how COVID-19 has impacted the position of migrant workers before discussing opportunities created for migrant workers and the way forward.

## The Impact of COVID-19 on Migrant Workers

According to the Migration DATA portal, one out of five workers in Northern America and Western and Middle European countries is a migrant worker. Sectors in which they are working, for instance, are agriculture, domestic work, cleaning services, distribution centers, and personal care workers. Migrants make important contributions in addressing the pandemic but are at the same time exposed to higher risks of contracting the virus (Fasini and Mazza 2020). Due to various reasons, labor migrants, especially those at the low end of the labor market, are among the first to be affected. They are disproportionately impacted by the COVID-19 pandemic as well as the measures taken to tackle it.

Working remotely and from home has been part of the lockdown in many countries. Since, immigrants are less likely to work in jobs that can be performed remotely and, consequently, were either exposed to an increased risk of being infected by the virus or suffered from loss of income if they discontinued working (Borjas and Cassidy 2020). Migrant workers often work for temporary work agencies and those with short-term contracts or working for such agencies were among the first to lose their jobs. They were often excluded from national COVID-19 policy responses such as compensation for loss of income, un-employment benefits, or social security (ILO 2020). In cases in which residency is linked to employment, this not only led to job loss but the loss of residency as well, putting migrant workers into undocumented or irregular status as travel restrictions and lack of spare funds to pay for traveling home prevented them from returning to their home countries. This equally occurred when visa or work permits expired during the coronavirus pandemic. Furthermore, travel restrictions prevented migrant workers from taking up employment abroad even if they had already signed a contract or had made expenses to a recruitment agency or facilitator, for instance. Information on COVID-19 measures is often only available online and not in a language migrant workers understand, making these measures less accessible for them. Finally, living conditions in crowded housing pose a particular risk to the spread of COVID-19 among migrant workers. They are often accommodated in abhorrent conditions with multiple persons in a room, making it impossible to keep the required distance to prevent infections. Accommodation provided by their employers or by a temporary work agency for whom they work creates multiple dependencies. Migrant workers who refused to share a room with a person whom they did not know got fired because of refusing. Thus, migrant workers are excluded by host societies, which is one of the shortcomings of the old common revealed by the COVID-19 crisis as identified by the editors of this book in Chap. 10.1007/978-3-030-65355-2_1 of The New Common.

## Responses on the Position of Migrant Workers

Paradoxically, sectors in which migrant workers work are characterized by unskilled labor that is looked down upon. However, during the coronavirus crisis, the work of migrant workers have been labeled as “essential jobs” (Fasini and Mazza 2020). Countries with a high dependency on migrant workers in these sectors came to realize the importance of these workers. Although they are praised for their work during the coronavirus crisis this has not led to an improvement of their working and living conditions or to more job security and higher wages.

Indeed, in many Western European countries, they were needed in seasonal agriculture to harvest the products. Confronted with the closing of borders, governments were faced with the ethical question to balance health concerns and economic losses, with the position of the migrant worker as the object to this dilemma. The economic argument prevailed; regardless of the travel bans, Italy flew in farmworkers from Morocco, and Germany flew in some 80,000 seasonal workers from Eastern Europe amid the corona crisis (Pettrachin 2020). The risks for the migrant workers seemed to be of subordinate importance. While it is well known that migrant workers are accommodated in crowded collective facilities, are collectively transported in vans to and from the workplace, and work in close proximity, these factors did not ring any alarm bells. Only when a new outbreak of COVID-19 emerged in the meat processing industry in Germany and the Netherlands, attention was paid to their living and working conditions. However, the concerns were not about the well-being of the migrant workers but about of the risk of the spread of the coronavirus, again demonstrating an assessment of the situation that balances health concerns with economic losses and indeed, a reflection of Hardin’s “Tragedy of the Commons” (Hardin 1968).

Despite the neglect of the position of migrant workers and mostly due to economic goals, the coronavirus crisis generated some positive responses that ameliorated the position of migrant workers. Italy provides such an example where the situation of undocumented migrant workers received the attention that led to an overall improvement—albeit on a temporary basis. After realizing that, during the lockdown and with borders closed, Italian farmers are highly dependent on undocumented migrant workers for harvesting, the migration skeptical Italian government adopted a law to temporarily regularize the status of up to 200,000 undocumented migrant workers to prevent the collapse of the agricultural sector. This exemplifies the balancing of public health and economic concerns with a recognition of the important role of migrant workers and an acknowledgment of the precarity of their situation. However, by June 15 2020, 32,000 applications were received primarily from domestic workers and caregivers (91%) and, surprisingly, not that many from farmworkers due to strict conditions and dependency on their employers to apply for regularization. Another positive approach is the regularization of migrants with pending immigration applications in Portugal, including access to health care and social services during the pandemic. Other examples of reckoning and improving the situation of migrant workers are the extension of migrant working visas or amnesties to alleviate constraints faced by migrant workers and their families in some destination countries, e.g., Belgium, Lebanon, Morocco, South Africa, Thailand, and the United Arab Emirates (ILO Policy Brief, April 2020).

## Revamping the Debate Post-Corona

These practices are part of a wider debate, not only in the EU but more broadly in migration destination countries, which is based on the paradox between, on the one hand, migration skepticism resulting in strict migration policies and a lack of legal migration pathways and, on the other hand, the demand for cheap labor of corporations to maximize profit at the expense of labor laws and humane living conditions. In the EU, the debate evolves around the balance between free movement and decent labor standards (Anderson and Ruhs 2010; Bogoeski 2020). The systemic and structural manifestation of the position of migrant workers points at a societal acceptance of different treatment that ties in with the current Black Lives Matter movement against discrimination of black people and those with a colored skin or migratory background, who often work in the same, low-skilled, low-paid sectors. The time seems to have arrived to have a broader discussion on inclusive societies and to curb latent forms of discrimination. This momentum should be used to regain attention for migrant workers in precarious work situations and to reconsider fundamental—social, legal, and economic—structures in our society that facilitate such situations. One thing we have learned from this crisis is that such a process gains wider attention if the economic and business argument could be integrated into this debate. Let us not lose this momentum and strive for a more inclusive society and curb inequality as encouraged by the sustainable development goals.
